# Pyrrole-Doped Polydopamine-Pyrrole (PDA-nPY) Nanoparticles with Tunable Size and Improved NIR Absorption for Photothermal Therapy

**DOI:** 10.3390/ph16121642

**Published:** 2023-11-23

**Authors:** Yuan He, Ziyang Li, Huiling Su, Yanan Sun, Wei Shi, Yunfeng Yi, Dongtao Ge, Zhongxiong Fan

**Affiliations:** 1 Department of Cardiothoracic Surgery, The 909th Hospital, School of Medicine, Xiamen University, Zhangzhou 363005, China; heyuan_1988@126.com; 2 Institute of Materia Medica & College of Life Science and Technology, Xinjiang University, Urumqi 830017, China; 3 The Higher Educational Key Laboratory for Biomedical Engineering of Fujian Province/Research Center of Biomedical Engineering of Xiamen, Xiamen Key Laboratory of Fire Retardant Materials/Fujian Provincial Key Laboratory of Fire Retardant Materials, Department of Biomaterials, College of Materials, Xiamen University, Xiamen 361005, China; 31420161150107@stu.xmu.edu.cn (Z.L.); 31420151150884@stu.xum.edu.cn (H.S.); sunyanan@xmu.edu.cn (Y.S.); shiwei@xmu.edu.cn (W.S.)

**Keywords:** polydopamine, pyrrole, self-oxidative polymerization, photothermal treatment, cancer therapy

## Abstract

Polydopamine (PDA) as a melanin-like biomimetic material with excellent biocompatibility, full spectrum light absorption capacity and antioxidation property has been extensively applied in the biomedical field. Based on the high reactivity of dopamine (DA), exploiting new strategies to fabricate novel PDA-based nano-biomaterials with controllable size and improved performance is valuable and desirable. Herein, we reported a facile way to synthesize pyrrole-doped polydopamine-pyrrole nanoparticles (PDA-nPY NPs) with tunable size and enhanced near-infrared (NIR) absorption capacity through self-oxidative polymerization of DA with PY in an alkaline ethanol/H_2_O/NH_4_OH solution. The PDA-nPY NPs maintain excellent biocompatibility and surface reactivity as PDA. By regulating the volume of added PY, PDA-150PY NPs with a smaller size (<100 nm) and four-fold higher absorption intensity at 808 nm than that of PDA can be successfully fabricated. In vitro and in vivo experiments effectively further demonstrate that PDA-150PY NPs can effectively inhibit tumor growth and completely thermally ablate a tumor. It is believed that these PY doped PDA-nPY NPs can be a potential photothermal (PT) agent in biomedical application.

## 1. Introduction

Polydopamine (PDA) as a melanin-like biomimetic material, can be fabricated via self-oxidative polymerization of dopamine (DA) in alkaline solutions to form PDA film or particles [1]. There exist abundant functional groups on the PDA surface including amino, catechol hydroxyl, and carbonyl, which provide PDA with some special properties such as excellent adhesion, metal ion chelation, redox activity, free radical quenching and full-spectrum light absorption characteristics [2,3,4,5]. Therefore, functional PDA coatings or particles have been widely applied in the field of energy batteries, environmental protection, drug delivery and disease diagnosis and treatment [6,7,8].

The self-oxidative polymerization of DA can be simply reacted in various classic systems, such as alkaline Tris-HCl buffer [9], NaOH solution [10,11], ethanol/ammonia solution [12,13], and alkaline carbonate buffer [14], without any additional oxidants assisting. However, the self-oxidative polymerization of DA in alkaline solutions is extremely complex, and the formation mechanism of PDA is not fully understood till now. Additionally, PDA is not a traditional polymer with a certain degree of polymerization. Various DA intermediate structures with high reactivity are formed during the oxidation of DA [15,16]. These reactive monomers will further form much DA-based or 5,6-dihydroxyindole (DHI)-based unit structures via covalent or non-covalent interactions, and finally form supramolecular polymer PDA through self-assembly [17,18]. It is noteworthy that the selected reaction solution, pH, DA concentration and reaction temperature and time will all affect the morphology and chemical structure of the obtained PDA, especially for the PDA particles [19,20].

The application of materials in the biomedical field has strict limitations on size. Compared with other reaction solutions, PDA nanoparticles (NPs) with tunable nanoscale size and dispersion stability could be synthesized more easily in an alkaline ethanol/ammonia/water solution at room conditions by just regulating the volume of added ammonia (as well as the pH) [12]. Even though a solution with higher pH is advantageous for preparing PDA with a smaller size, the absorption ability of PDA NPs in the NIR region and production yield will be weakened [21]. Nowadays, some new strategies using a third component to control the polymerization of PDA to reduce size have been reported [22,23,24,25]. Some molecules, such as edaravone [24] (a strong free radical scavenger), boronic acids and Lewis base [25], can inhibit the polymerization of DA to reduce the size, but the final product yield also decreases. Other molecules can promote DA oxidation, such as some stable free radicals (PTIO·) [24], and increase the number of PDA seed formation to reduce the size without affecting the yield. Actually, adding molecules that can interact with DA into the DA self-oxidation reaction system will have an effect on the particle size, light absorption, and redox activity of the PDA [26,27,28].

Photothermal therapy is a non-invasive cancer therapeutic approach that can achieve local targeted hyperthermia of cancer tissue by using photothermal agents (PTAs) and suitable laser irradiation. The photothermal conversion capacity of the PTAs plays a very important role. Therefore, developing efficient PTAs is presently a major research topic [29,30]. Polypyrrole (PPY) is a kind of conjugated polymer with outstanding photothermal properties [31]. Compared with PDA, PPY has better absorption capacity in the NIR region and is regarded as a potential high-efficiency PTA. Nowadays, some DA-PY copolymers exhibiting improved optical and electrical properties have been reported [32,33]. However, due to the high oxidation potential of pyrrole (PY) for polymerization, all reported DA-PY copolymer fabrication methods require the addition of exogenous oxidants (Fe^3+^, persulfate), which greatly increase the difficulty in regulating the size and morphology of obtained production suitable for biomedical application.

In this work, we successfully synthesized pyrrole-doped polydopamine nanoparticles (PDA-nPY NPs) with regulated size and enhanced light absorption in the near-infrared (NIR) region via one-pot self-oxidative polymerization in an improved ethanol/NH_4_OH/water solution. The reaction needs no requirement of additional oxidants or template molecule assistance to prepare unique spherical particles with nanoscale. The effect of added PY on the size distribution, morphology and chemical structure of PDA-nPY NPs was studied to investigate the possible interaction between DA and PY. Taking advantage of the enhanced NIR absorption ability of PDA-nPY NPs, we further investigated the photothermal therapeutic capacity of PDA-150PY NPs against tumors.

## 2. Results and Discussion

### 2.1. Synthesis and Characteristics of PDA-nPY NPs

To synthesize the PDA-PY NPs, we adopted an improved classical reaction system of H_2_O/ethanol/NH_4_OH mixed solution, in which the volume ratio was changed from 9:4:0.2 to 9:14:0.2. The mixed solution with new ratio was conducive to fabricating NPs with stable and smaller sizes (<200 nm). Before polymerizing in the H_2_O/ethanol/NH_4_OH solution, DA and PY were first premixed in pure water for 2 h under vigorous stirring, and then the premixture of DA with different volumes of PY was added into the H_2_O/ethanol/NH_4_OH solution and proceeded to a self-oxidative reaction with constant stirring without any other oxidants added for 24 h. The final products were collected via ultrafiltration interception and recorded as PDA-nPY (n meaning the added volume of PY). Considering the limited solubility of PY monomers in water, the highest added volume of PY was 180 μL, which was not fully soluble in the premixed solution.

**Figure 1:**
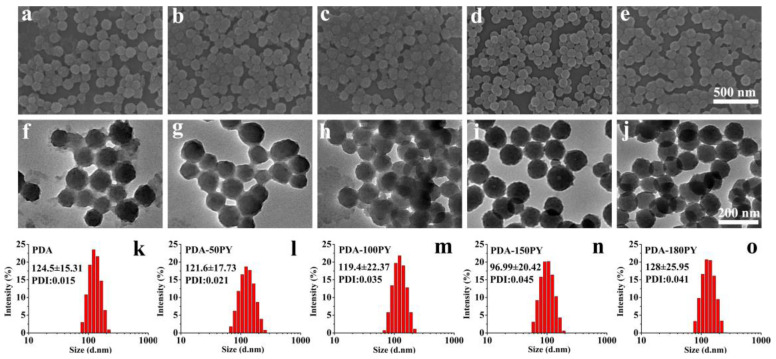
SEM images, TEM images and size distributions of PDA-nPY NPs: PDA (**a**,**f**,**k**), PDA-50PY (**b**,**g**,**l**), PDA-100PY (**c**,**h**,**m**), PDA-150PY (**d**,**i**,**n**) and PDA-180PY (**e**,**j**,**o**).

**Figure 2:**
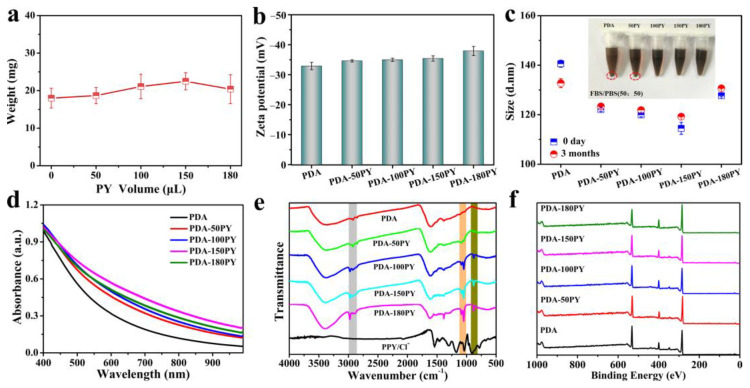
(**a**) Production weight of PDA-nPY. (**b**) Zeta potential measurement of PDA and PDA-nPY NPs in water. (**c**) Size stability of PDA and PDA-nPY NPs dispersed in water for three months, and photograph of dispersion stability of PDA and PDA-nPY NPs suspended in FBS/PBS (50:50) for 48 h (insert, the red circle indicating the precipitations in PDA and PDA-50PY). (**d**) UV-VIS absorbance spectra of PDA and PDA-nPY NPs in water at a concentration of 50 μg/mL. (**e**) FTIR spectra of PDA, PDA-nPY and PPY/Cl^−^. (**f**) XPS survey spectra of PDA and PDA-nPY.

The SEM image shows that the synthesized PDA-nPY NPs had a homogeneous spherical morphology similar to PDA (Figure 1). Moreover, when increasing the amount of PY, the surface of PDA-nPY NPs became smoother. As the TEM images show, some irregular stackings could be observed around PDA NPs, while PDA-nPY NPs had clear surfaces with almost no adhesion between NPs. The size distribution indicated that PDA and PDA-nPY NPs with high uniformity size can be synthesized in this improved solution, and the added PY did not significantly affect the morphology and size. The hydrodynamic diameter of PDA and PDA-nPY NPs were all approximately 120 nm; the more PY was added, the smaller the size appeared. The PDA-150PY NPs had the smallest size with less than 100 nm, indicating that appropriately increasing the added volume of PY is beneficial for reducing particle size. When the added PY exceeded the solubility limitation, the size regulatory effect of PY was also inhibited. Moreover, the addition of PY had no effect on the production of final obtained PDA-nPY under the same concentration of DA, which indicated that the PY had no inhibition on the DA self-oxidative polymerization (Figure 2a).

The obtained PDA-nPY NPs had the same excellent water dispersibility as PDA NPs, and they could not all be completely precipitated from the reaction solution even when the centrifugal force was above 22,000 g. It was found that the tested Zeta potential of PDA and PDA-nPY NPs in water were all <−30 mV (Figure 2b), which afforded PDA-nPY NPs with excellent suspension stability. Therefore, PDA and PDA-nPY NPs could be stably suspended in water for three months without significant size changes or precipitation. Furthermore, PDA-nPY NPs also presented sufficient suspension stability in FBS/PBS without any surface modification (Figure 2c). PDA-nPY NPs with more than 100 μL PY added could be stably suspended in FBA/PBS for 48 h without aggregation and precipitation. The surface negativity of PDA was derived from the surface exposed hydrophilic functional groups including amino and catechol hydroxyl groups. After PY was added, the Zeta potential of PDA-nPY showed enhanced negativity. We speculated that there might be more amino and catechol hydroxyl groups on the surface of PDA-nPY than of PDA.

Similar to PDA, PDA-nPY NPs performed broad light absorption ability in the UV-VIS-NIR region (Figure 2d). Moreover, the PY doped PDA-nPY all had enhanced light absorption capacity in the NIR region than that of PDA, and PDA-150PY NPs had the highest light absorption capability at 808 nm (the most commonly used wavelength in the first NIR region for photothermal treatment), which was four-folds higher than that of PDA at the same concentration. 

Subsequently, the chemical structure of PDA-nPY was analyzed by FTIR and XPS. Comparing FTIR spectra of PDA, PPY/Cl^-^ and PDA-nPY (Figure 2e), it was found that the FTIR spectra of all PDA-nPY had high similarity with that of pure PDA, while also possessing several characteristic peaks of PPY/Cl^−^. The peak at 3400 cm^−1^, a broad absorption band, was attributed to the stretching vibration of N-H and aromatic C–OH. And the peak at 1620 cm^−1^ corresponded to the stretching vibration of C=C of the PDA ring [15]. Meanwhile, the typical absorption peaks of PY could also be seen; 1040, 1090 and 875 cm^−1^ were assigned to =C–H in-plane vibrations, N–H in-plane deformation and -C–H bending vibration of the PY ring, respectively. Furthermore, with increasing amounts of PY added, the peak intensity around 2950 cm^−1^ (aliphatic C–H stretching vibrations), 1040, 1090 and 875 cm^−1^ increased obviously [33,34]. Next, the chemical composition of PDA and PDA-nPY were investigated by XPS measurement. XPS survey spectra (Figure 2f) indicated that PDA and PDA-nPY had the same basic elements including C, N and O. Table 1 lists in detail the element contents and N/C ratios calculated from XPS survey spectra. Compared with PDA, the N/C ratio in PDA-nPY samples increased over PDA, mainly due to the addition of PY with a higher N/C ratio (N/C in PY = 0.25, N/C in DA = 0.125). The element changes suggest that PY was successfully involved in the formation of the final product. However, it was noted that the N/C ratios in all PDA-nPY were still close to that of PDA, indicating that the main component of PDA-nPY was DA.

**Figure 3:**
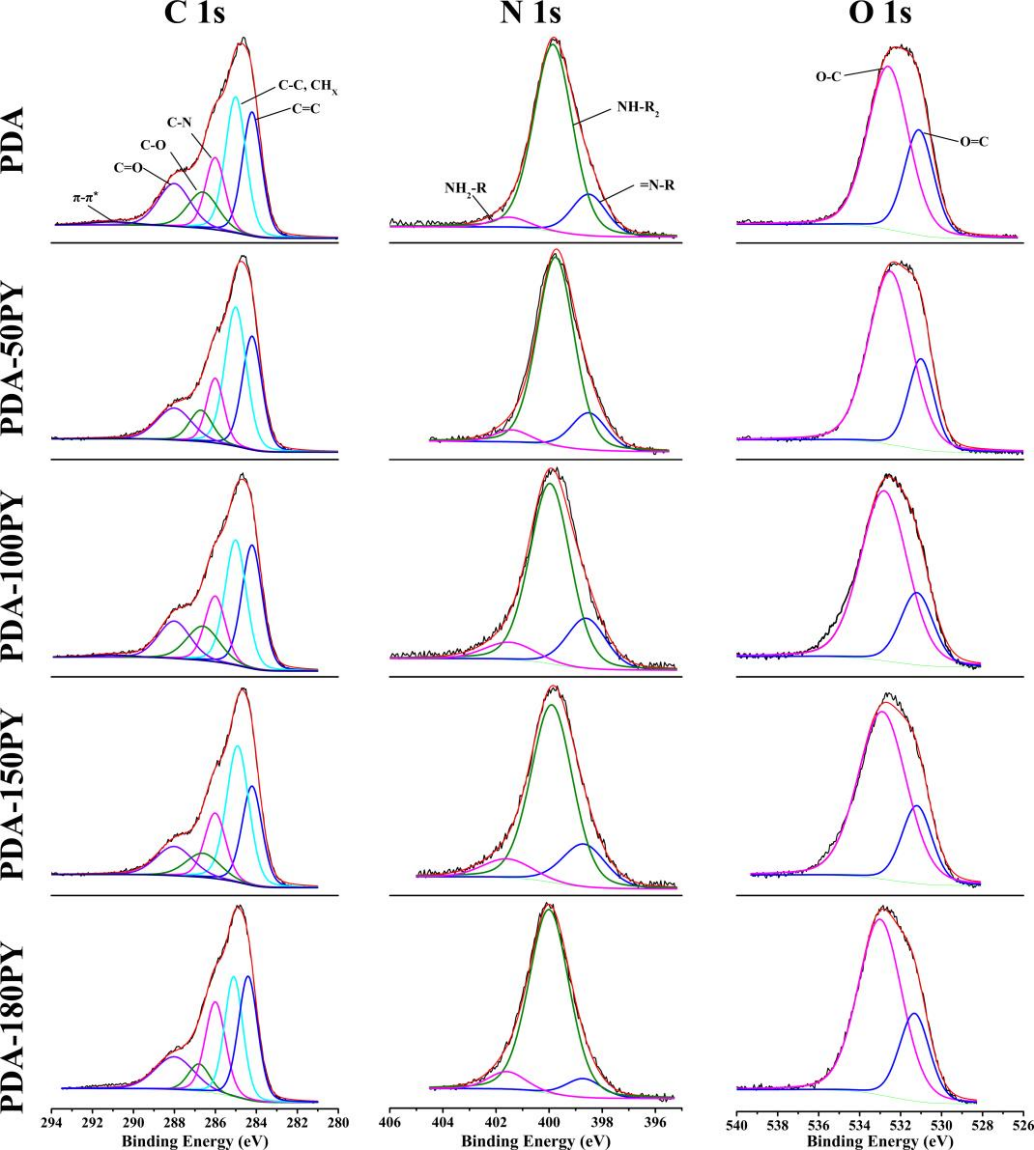
High-resolution XPS spectra of C1s, N1s and O1s of PDA and PDA-nPY.

To further prove the detailed chemical structure of PDA-nPY, high-resolution spectra of C1s, N1s and O1s of PDA and PDA-nPY were examined (Figure 3) [35,36]. The C1s, N1s and O1s spectra of PDA and PDA-nPY could be curve fitted with the same chemical bonds but the proportions were different. The percentage contribution of each functional group in the C1s, N1s and O1s regions are listed in Table 2. The C1s region was fit with six peaks assigned to C=C, CHx/C–C, C–N, C–O, C=O and π-π*. The N1s region was fit with three peaks assigned to =N-R, NH-R_2_ and NH_2_-R, and the major structure of N in both PDA and PDA-nPY were secondary amine (NH-R_2_). The O1s region was fit with two peaks assigned to O=C and O–C, and the catechol group (O–C) content was obviously higher than the quinone form (O=C) in both PDA and PDA-nPY. 

Compared with PDA, the proportion of primary amine structure (NH_2_-R) belonging to uncyclized DA in PDA-nPY samples were obviously increased. Meanwhile, the proportion of oxidized quinone structure (O=C) in PDA-nPY were significantly lower than that in PDA. Undoubtedly, the increasing contents of hydrophilic groups including -NH_2_ and -OH in PDA-nPY led to a more negative surface potential and suspension stability of PDA-nPY than that of PDA. Additionally, it also indicated that the self-oxidation and intramolecular cyclization of DA were inhibited in the DA and PY coexisted reaction solutions.

### 2.2. DPPH Radical Scavenging Capacity of PDA-PY

PDA has excellent antioxidant capacity and can effectively quench free radicals. Previous research has demonstrated that, compared with PDA, reduced PDA contains abundant catechol groups with enhanced antioxidant properties [4]. The above XPS results show that the content of reduced -OH in PDA-150PY increased to 77.47%, which was higher than that in PDA (68.29%). We speculated that the PDA-150PY NPs would have excellent antioxidant capacity. To verify this hypothesis, the DPPH radical scavenging by PDA-150PY NPs was measured.

**Figure 4:**
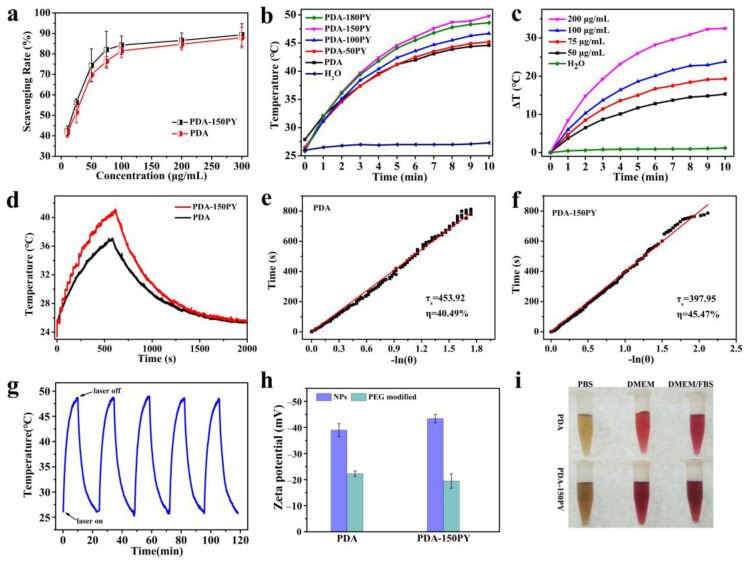
(**a**) DPPH radical scavenging rate of PDA and PDA-150PY NPs with different concentrations. (**b**) Photothermal curves of PDA and PDA-nPY NP solutions with the same concentration of 100 μg/mL under 808 nm laser irradiation (1.5 W/cm^2^) for 10 min. (**c**) Photothermal curves of PDA-150PY NPs in water at different concentrations irradiated by 808 nm laser (1.5 W/cm^2^) for 10 min. (**d**) The temperature change curve of PDA and PDA-150PY NPs within a heating–cooling cycle. (**e**) The time plot of the negative natural logarithm of temperature of PDA NPs during the cooling process. (**f**) The time plot with the negative natural logarithm of temperature of PDA-150PY NPs during the cooling process. (**g**) Temperature change curves of PDA-150PY NPs in water (100 μg/mL) under 808 nm laser irradiation (1.5 W/cm^2^, 10 min) for five cycles. (**h**) Zeta potential changes of PDA and PDA-150PY NPs after surface modification. (**i**) Photograph of PDA-PEG and PDA-150PY-PEG NPs suspended in PBS, DMEM and DMEM/FBS.

The result in Figure 4a showed that both PDA and PDA-150PY NPs could effectively scavenge DPPH radicals in a dose-dependent manner, and under the same incubated concentration, the radical scavenging capacity of PDA-150PY was slightly better than that of the PDA NPs.

### 2.3. The Reaction Mechanism of DA–PY

**Scheme 1:**
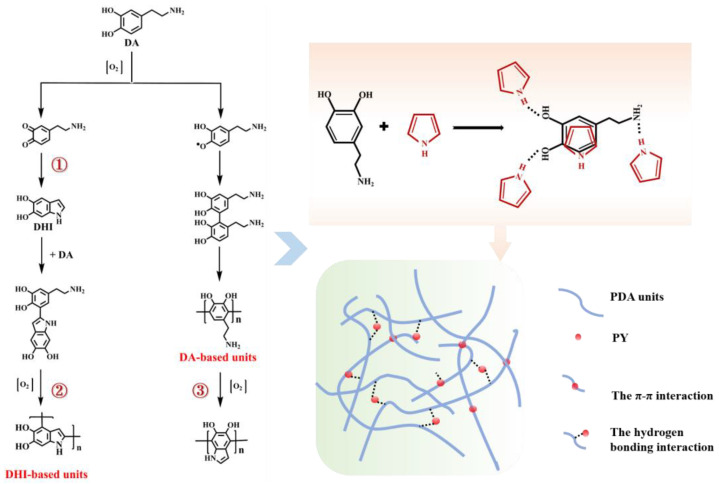
Schematic illustration of the possible reaction mechanism and chemical structure of PDA-nPY.

In the DA and PY coexisted self-oxidation reaction system, the added PY could regulate the size and light absorption in the NIR region of the obtained product. Chemical structure investigations further confirmed PY could be doped into PDA during the self-polymerization of DA in an alkaline H_2_O/ethanol/NH_4_OH solution. Meanwhile, due to the harsh polymerization conditions of PY, it was impossible to generate PPY in these alkaline self-oxidation conditions [37]. Therefore, the production of PDA-nPY did not decrease with the addition of PY compared with PDA, indicating that the added PY did not inhibit the polymerization of DA. However, the results of XPS showed that the content of DA-based units in PDA-nPY increased, illustrating that the addition of PY inhibited the intramolecular oxidation and cyclization of DA (such as step ①–③ in Scheme 1). 

Considering that it was impossible to generate PPY from PY with just dissolved oxygen in a short time and also had no active groups which could directly and covalently react with DA via Michael addition/Schiff base reactions, we speculated that the possible interaction between PY and DA in this self-oxidative system was through π-π stacking or hydrogen bond interaction during the pre-mixing stage [17,38]. However, the presence of intermolecular hydrogen bonds might bring steric hindrance to followed intramolecular self-oxidative cyclization of DA, which is beneficial for a more open-chain DA-based structure in the PDA-nPY formation. Actually, the PY acted as a dopant to regulate and optimize the morphology and light absorption capacity of obtained PDA-nPY. As an electron-rich nitrogen heterocyclic structure, PY could form a charge transfer complex with the nearest DA oligomers or short PDA chains via π-π interaction or hydrogen bonding, thereby enhancing the intermolecular electron transfer between PDA chains and improving the absorption performance of PDA-nPY in the NIR region.

### 2.4. In Vitro Biocompatibility and Photothermal Effect

PDA has been demonstrated to be a potential PTA with excellent biocompatibility [39]. Based on the above improved absorption in the NIR region of PDA-nPY NPs, the photothermal performance of PDA-nPY NPs under 808 nm laser irradiation was investigated. PDA and PDA-nPY NP solutions with the same concentration (100 μg/mL) were irradiated by 808 nm laser (1.5 W/cm^2^) for 10 min. It could be seen that all PDA and PDA-nPY NP solutions could rise above 43 °C, which is high enough to cause substantial thermal damage on tumors within 10 min of irradiation (Figure 4b). The PY-doped PDA-nPY NPs performed enhanced photothermal performance more than PDA, especially the PDA-150PY NPs, which had the best NIR light-induced thermal ability with more than 7 °C higher than that of PDA solution. Furthermore, the photothermal performance of PDA-150PY NPs also performed a concentration dependence (Figure 4c). The photothermal conversion efficiency (ŋ) of PDA and PDA-150PY NPs were further calculated, respectively (Figure 4d–f). It could be seen that the photothermal conversion efficiency of PDA-150PY was 45.47%, which was higher than that of PDA. Therefore, the doped PY effectively improved the absorption capacity in the NIR region as well as the photothermal conversion performance of PDA-150PY compared with PDA.

In addition, the PY-doped PDA-150PY NPs also presented excellent photostability. After five cycles of on–off laser irradiation, the PDA-150PY NP solution could reach almost the same temperature, indicating that the light-induced temperature rising ability of PDA-150PY was not affected and it had good photothermal stability (Figure 4g).

The excellent photothermal capacity and photostability confirmed that the PDA-150PY NPs can also be used as a potential PTA. In order to improve its stability of long circulation in vivo and tumor-targeting capability, the surface of PDA-150PY NPs were further covalent modified with PEG-NH_2_. After modification by PEG-NH_2_, the negativity of the Zeta potential decreased (from <−40 mV to −22 mV) and the difference of surface Zeta potential between PDA and PDA-150PY was eliminated (Figure 4h). PDA-PEG and PDA-150PY-PEG NPs both performed excellent suspension stability in various solutions (Figure 4i).

**Figure 5:**
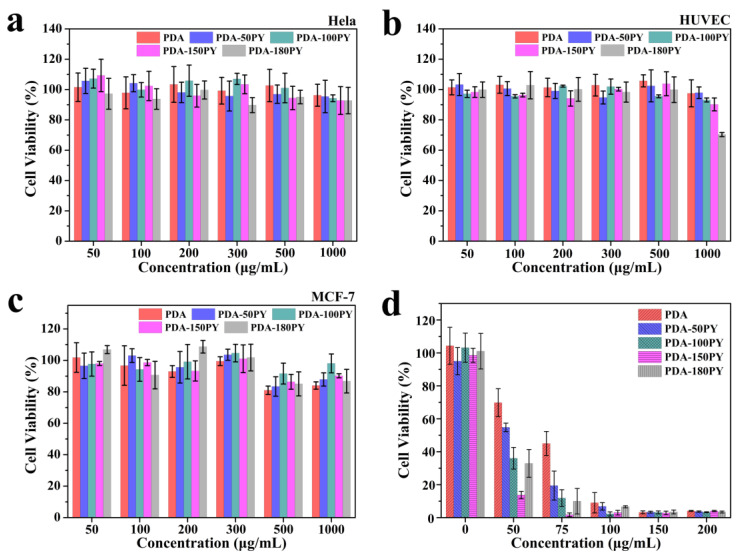
Cell viability of Hela (**a**), MCF-7 (**b**) and HUVEC (**c**) co-incubated with various concentrations of PDA-PEG and PDA-nPY-PEG NPs for 24 h. (**d**) Cell viability of MCF-7 treated with different concentrations of PDA-PEG and PDA-nPY-PEG NPs irradiated by 808 nm laser (1.5 W/cm^2^) for 10 min.

To evaluate the cytotoxicity of PDA-nPY NPs, Hela, MCF-7 and HUVEC cells were incubated with different concentrations of PDA-PEG and PDA-nPY-PEG NPs and tested by a standard MTT assay; the PDA NPs were used as the control group. The results demonstrate that almost all PDA-nPY NPs showed good biocompatibility even at a higher concentration of 1000 μg/mL against no matter cancer cell lines (Hela and MCF-7) or normal cell line (HUVEC) without laser irradiation, in which the cell viability was maintained over 80% ability (Figure 5a–c). 

The in vitro photothermal cytotoxicity of PDA-nPY NPs against MCF-7 cells was further investigated under 808 nm laser irradiation (1.5 W/cm^2^, 10 min). Only laser irradiation without PTAs had no effect on MCF-7 cell viability. When co-cultured with the same concentration of NPs under irradiation, the photothermal lethality of all PDA-nPY-PEG NPs against MCF-7 was higher than that of PDA-PEG NPs, and PDA-150PY-PEG NPs performed the best photothermal mortality on MCF-7 cells (Figure 5d). For instance, at the same concentration of 50 μg/mL, PDA-150PY-PEG could kill more than 80% of MCF-7 cells under irradiation, while the cell viability of the PDA-PEG treated group was still above 70%. To achieve the same photothermal lethality effect as 50 μg/mL of PDA-150PY-PEG, the concentration of PDA-PEG NPs needed to exceed 100 μg/mL.

### 2.5. In Vivo Photothermal (PT) Treatment Performance

In vitro experiments confirmed PDA-nPY NPs have better photothermal performance than PDA, and PDA-150PY had the highest photothermal effect. Therefore, we chose PDA-150PY-PEG to investigate the in vivo thermally ablate tumor effect against the MCF-7 tumor model. Same doses (300 μg) of PDA-PEG and PDA-150PY-PEG NPs dispersed in PBS were intravenously injected into MCF-7 tumor-bearing mice, and the control group were injected with equal volumes of PBS. Twelve h post-injection, the tumor site of all mice was irradiated with 808 nm laser irradiation for 10 min (1.5 W/cm^2^) per day, and the photothermal treatment was continued for four days. 

**Figure 6:**
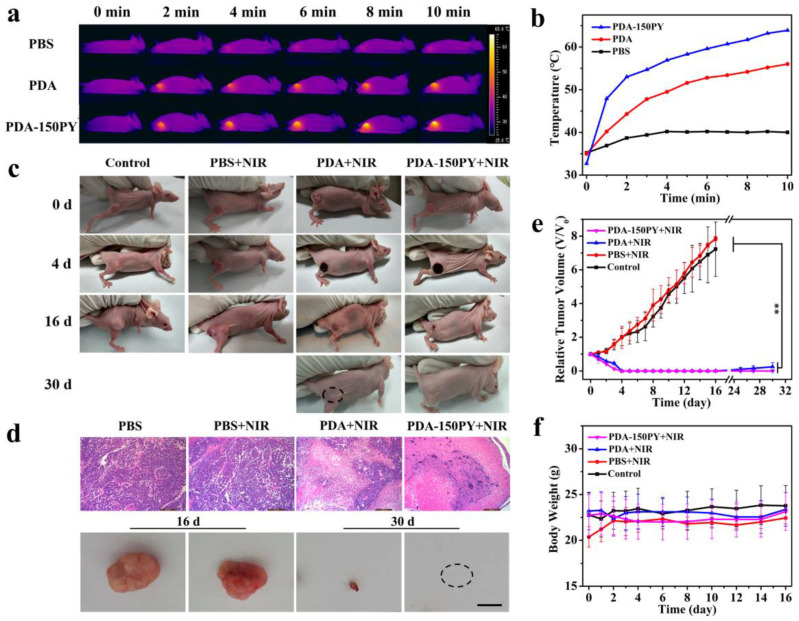
(**a**) IR thermal images of MCF-7-bearing nude mice under 808 nm laser irradiation (1.5 W/cm^2^) for 10 min at every 2 min time point. (**b**) Tumor-site temperature curves of mice irradiated by 808 nm laser (1.5 W/cm^2^) for 10 min after 24 h injection of PDA-PEG and PDA-150PY-PEG. (**c**) Photographs of mice with PT treatment on day 0, 4, 16 and 30. (**d**) H&E staining slices of tumor-site in four groups after PT treatment and photographs of tumor tissues after photothermal treatment (scale bar = 1 cm). (**e**) The related tumor volume in four groups with PT treatment. (**f**) Body weight curves of four mice groups with PT treatment. *p* values were calculated via ANOVA with Turkey’s post-hoc test: ** *p* < 0.01.

The temperature changes of the tumor site injected with different NPs reflected that the tumor temperature in the PBS-treated group increased only 4 °C, which could not cause effective anti-tumor hyperthermia on MCF-7 tumor tissue. For mice injected with PDA-150PY-PEG NPs, the tumors’ temperature quickly rose to above 50 °C within 2 min, and then remained at a high level above the effective thermal damage temperature within 10 min of irradiation. The temperature of PDA-PEG-injected mice raising to 50 °C need approximately 5 min and the final temperature was near 55 °C under irradiation (Figure 6a,b). The results demonstrate that PDA-150PY-PEG could generate more adequate heat than PDA-PEG to achieve photothermal ablation on tumor tissue under the same irradiation conditions. The H&E staining images of tumor tissues after one irradiation treatment with different NPs further demonstrated that only laser irradiation under experimental conditions did not have side effects on tumor tissue. In contrast, obvious tissue necrosis appeared in the tumor tissue with PDA-PEG and PDA-150PY-PEG NP-treated groups after irradiation, especially for the PDA-150PY-PEG NP-treated groups (Figure 6c,d).

MCF-7 tumor-bearing mice were divided into four group (PBS, PBS + NIR, PDA-PEG + NIR, PDA-150PY-PEG + NIR) and treated with a photothermal treatment for four consecutive days. The digital photos and tumor volume changes of mice were recorded during the whole treated procedure (Figure 6c,e). Compared with the control group (only PBS), 1.5 W/cm^2^ laser irradiation alone had no inhibition effect on tumor growth. After four days of treatment, the tumor tissues in both PDA-PEG and PDA-150PY-PEG-treated groups were visibly ablated, and black scabs caused by tissue necrosis appeared. On the 16th day after treatment, black scabs began to fall off and the tissue was recovering well. But from the 24th day, half of the mice in the PDA-PEG + NIR group (two out of four mice) had tumor recurrence at the treatment site, while no tumor recurrence appeared in PDA-150PY-PEG-treated mice. This result proved that PDA-150PY-PEG had better photothermal therapeutic effect than conventional PDA-PEG NPs.

**Figure 7:**
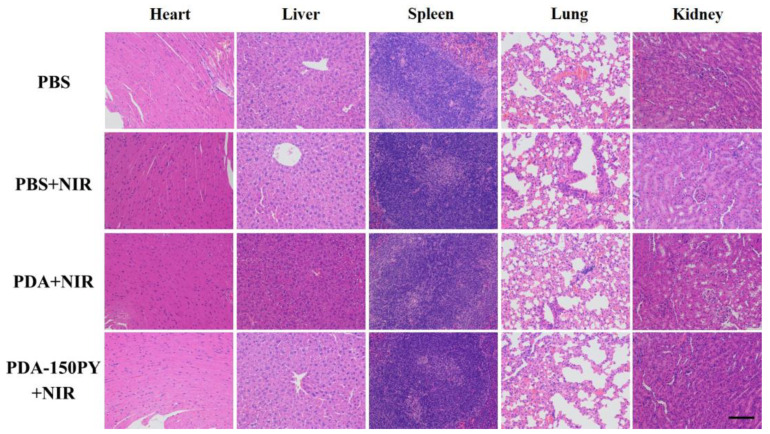
H&E staining of main organs with different treatments. (Scale bar = 200 µm).

Furthermore, the body weight of all mice during the whole experimental period had no significant decrease in all four groups (Figure 6f). The H&E staining results of the main organs (including heart, liver, spleen, lungs and kidneys) in each groups had no observable morphological damage (Figure 7), implying that the PDA-150PY-PEG had no evident toxicity nor side effects on the mice’s normal physiological activities and had good biosafety.

## 3. Materials and Methods

### 3.1. Materials

Dopamine hydrochloride (DA) was purchased from Sigma-Aldrich Co. Pyrrole (PY), ethanol, and ammonia (28–32%) were obtained from Sinopharm Chemical Reagent Co., Ltd., (Shanghai, China), and 1-ethyl-3-(3-dimethylaminopropyl) carbodiimide hydrochloride (EDC) and N-hydroxysuccinimide (NHS) were purchased from J&K Scientific Ltd. (Beijing, China). Amine-PEG-Amine (NH_2_-PEG-NH_2_, 6 kDa, ≥99.0%) was purchased from Chemgen Pharma Co., Ltd. (West Bengal, India), and 2,2-biphenyl-1-picrylhydrazyl (DPPH) was purchased from Aladdin Co., Ltd (Shanghai, China). All reagents were used directly without further purification. MCF-7, Hela and HUVEC cell lines were purchased from the Wuhan Cell bank of Chinese Academy of Sciences.

### 3.2. Synthesis of PDA-nPY NPs

PDA-nPY NPs were synthesized using a typical reaction method reported before with slight adjustment. In brief, 200 μL ammonia aqueous solution (28–30%), 4 mL ethanol and 15 mL deionized (DI) water was mixed with mildly magnetic stirring at ambient temperature for 30 min. A total of 50 mg dopamine hydrochloride (Sigma-Aldrich, Burlington, MA, USA) and different amounts of pyrrole (0, 50, 100, 150, and 180 μL) were premixed in 2 mL deionized water for 2 h and then dropwise injected into the above solution. Considering the low solubility of PY in water, the maximum amount of PY was limited at 180 μL. The reaction proceeded for 24 h. The final product was purified and collected successively by dialysis and filtrated centrifugation with 10 kDa (MWCO) ultrafiltration centrifuge tube to remove the solvent and oligomers. The obtained NPs were redistributed in ultrapure water. The concentration of PDA-nPY and PDA was measured by mass difference method after solvent evaporation.

PDA-nPY NPs were covalently modified with NH_2_-PEG-NH_2_ (MW = 6 kDa). An amount of 60 mg NH_2_-PEG-NH_2_ and 5 mL PDA-nPY dispersion (2 mg/mL) were added in 8 mL deionized water together and fully mixed under magnetic stirring for 2 h. Then, 80 μL ammonia was dropwise added in the above mixture (pH = 10) and continuously stirred for 12 h. The PEG-modified PDA-nPY was purified with an ultrafiltration centrifuge tube (10 kDa MWCO) to remove excess PEG. After purification by centrifugation and washing with deionized water, the sample was redistributed in deionized water and stored at 4 °C for the subsequent experiment. 

### 3.3. Characterization

The morphology was measured by scanning electron microscope (SEM, SU-70, Hitachi, Tokyo, Japan) and transmission electron microscope (TEM, FEM1400, JEOL Corp., Tokyo, Japan). The accelerating voltage of the SEM was 5.0 kV, and the TEM was running at 100 kV. The chemical structure of PDA-nPY was analyzed using Fourier transform infrared spectroscopy (FTIR, Nicolet IN 10, Thermo Fisher, Waltham, MA, USA), and the samples were recorded with KBr pressed pellet. The hydrodynamic diameters and Zeta potentials were measured by DLS (Nano ZS90, Malvern Instruments, Westborough, MA, USA). The UV-vis absorbance spectra of PDA-nPY were tested by UV-VIS spectrophotometer (UV-1750, SHIMADZU, Kyoto, Japan). The chemical composition of PDA-nPY was analyzed by X-ray photoelectron spectroscopy (XPS, ESCALAB 250, Thermo Scientific, Waltham, MA, USA). The excitation source was a monochromatic Al Kα X-ray operating at 15 kV and 150 W.

### 3.4. DPPH Radical Scavenging Test

DPPH was dissolved in ethanol at a concentration of 0.1 mM. PDA and PDA-150PY NPs were diluted to different concentrations ranging from 10 to 300 μg/mL with ethanol. Same volumes of DPPH solution and PDA or PDA-150PY NP solutions were mixed in a 96-plate and co-incubated in the dark for 30 min, and the absorbance at 517 nm was tested. Each sample was set with 4 parallels. The DPPH radical scavenging ratio was calculated by the equation: radical scavenging ratio = [1 − (Ai − Aj)/Ac] × 100%, in which Ai is the absorbance of different concentrations of NP samples mixed with DPPH solution, Aj is the absorbance of different concentrations of NPs mixed with ethanol, and Ac is the absorbance of DPPH solution mixed with ethanol.

### 3.5. Photothermal Performance of PDA-nPY NPs

PDA-nPY NPs were dispersed in DI water at a concentration of 100 μg/mL and placed in a quartz cuvette. Samples were vertically irradiated by 808 nm laser for 10 min at a power density of 1.5 W/cm^2^, and the temperature was monitored every 1 min using a digital thermocouple probe. The temperature changes of PDA-150PY NPs at different concentrations (50, 75, 100, and 200 μg/mL) were further measured with a power density of 1.5 W/cm^2^ 808 nm laser irradiation for 10 min. DI water was regarded as the control group.

The photothermal conversion efficiency of PDA-150PY was calculated according to following equations [40]:

(1) $$\eta =\frac{hA\Delta T_{max}-Q_{s}}{I\left(1-10^{-A_{808}}\right)}$$

(2) $$hA=\frac{mC}{\tau_{s}}$$

(3) $$\tau_{s}=\frac{-ln(\frac{T_{t}-T_{surr}}{T_{max}-T_{surr}})}{t}$$

where the *T_max_* is the maximum reached temperature of the tested solution, *T_surr_* is the ambient temperature, *T_t_* is the temperature of the tested solution at different time points, and the ∆*T_max_* = *T_max_* − *T_surr_*. *Q_s_* is the heat absorbed by the water. *I* is the power density of the laser. A808 is the absorption value of different tested solutions at 808 nm. *C* is the specific heat capacity of water.

### 3.6. Cell Culture

The human breast cancer cell line (MCF-7), human uterine cervix carcinoma cell line (Hela), and human umbilical vein endothelial cell (HUVEC) were cultured in DMEM-H medium supplemented with 10% fetal bovine serum (FBS, GIBCO) and 1% penicillin and streptomycin (Hyclone,) at 37 °C under a humidified 5% CO_2_.

### 3.7. In Vitro Cytotoxicity Assay

The cytotoxicity of PDA-nPY NPs was analyzed based on HUVEC, MCF-7 and Hela cells. Three cell lines were first separately seeded in a 96-well plate at a density of 8 × 10^3^ cells per well and cultured overnight. Then, a complete medium containing different concentrations of PDA-nPY NPs ranging from 50 μg/mL to 1000 μg/mL was added into each well and incubated with cells for another 24 h at 37 °C. The standard MTT assay was measured to determine the cell viabilityof-. The tested absorbance was at 570 nm with a microplate reader (Infinite M200 pro, TECAN, Männedorf, Switzerland).

### 3.8. In Vitro Photothermal Killing of Cancer Cells

To evaluate the photothermal killing effect of PDA-nPY on cancer cells, MCF-7 cells were seeded in a 96-well plate at a density of 8 × 10^3^ cells per well and cultured overnight; then, fresh medium containing different concentrations of PDA-nPY NPs ranging from 0 to 200 μg/mL was added and co-incubated with cells for another 4 h, and irradiated by 808 nm laser for 10 min at a power density of 1.5 W/cm^2^. After another 24 h culture, the relative cell viabilities were tested by the MTT assay.

### 3.9. In Vivo Photothermal Treatment for Tumor Ablation

Female nude mice 8–10-weeks old with an average weight of 20 g were purchased from Xiamen University Laboratory Animal Center. The whole animal experiments were completed in the Animal Center and followed the Chinese National Institutes of Health guidelines for care and use of laboratory animals.

An MCF-7 tumor model was built by subcutaneous injection of 100 μL of MCF-7 cells (5 × 10^6^ cells) into the right back of nude mice. Once the average volume of the tumors reached approximately 200 mm^3^, the mice were randomly divided into 4 groups with 5 mice in each group. The four group mice were intravenously injected with 100 μL of PBS, PDA-PEG and PDA-150PY-PEG only once and treated with: (1) PBS only; (2) PBS with 808 nm laser; (3) PDA-PEG NPs with 808 nm laser; and (4) PDA-150PY-PEG NPs with 808 nm laser. The injection dosage of PDA and PDA-150PY NPs was both 300 μg. After 12 h post-injection, the tumor site of each mouse was irradiated by 808 nm laser at a power density of 1.5 W/cm^2^ for 10 min per day and continuous irradiation for 4 days. On the first day of laser irradiation treatment, the in vivo thermal imaging was performed simultaneously using an IR thermal camera. The tumor volumes were measured daily, and the body weight was weighed the next day. The tumor volume was calculated according to the formula: Volume (mm^3^) = Length (mm) × Width^2^ (mm^2^)/2. 

After 2 days of photothermal treatment, a mouse was randomly selected from each group for dissection. Heart, liver, spleen, lung, kidney and tumor tissue were collected and fixed with fixative. The fixed tissue specimens were further stained with hematoxylin and eosin (H&E) and examined by light-field microscope.

## 4. Conclusions

In summary, we demonstrated that PY-doped PDA-nPY NPs had tunable size and improved absorption capacity in the NIR region and dispersion stability by regulating the added volume of PY. DA and PY had a similar reaction to DA self-oxidative polymerization in alkaline ethanol/H_2_O/NH_4_OH solution without the need for other oxidants. The added PY directly participated in the formation of final products and reduced the size of PDA-nPY without inhibiting the polymerization of DA and affecting the product yield. PY-doped PDA-nPY maintained excellent biocompatibility as PDA, and the enhanced NIR absorption capacity enabled them improved photothermal efficiency compared with PDA NPs. In vitro and in vivo experiments demonstrated that PDA-150PY, having the smallest size and best absorption capacity among the PDA-nPY NPs, could effectively and thoroughly ablate the tumor. We believe that this work can provide a green and facile strategy for fabrication of novel PDA-based nano-biomaterials with better performance.

## Figures and Tables

**Table 1:** Element content and ratios of PDA and PDA-nPY calculated from XPS survey spectra.

	C/%	N/%	O/%	N/C
**PDA**	67.5	9.34	23.16	0.138
**PDA-50PY**	68.14	9.48	22.38	0.139
**PDA-100PY**	68.58	10.01	21.41	0.146
**PDA-150PY**	70.08	9.92	20.01	0.142
**PDA-180PY**	70.72	10.7	18.58	0.151

**Table 2:** Concentration of XPS functional groups in PDA and PDA-nPY.

Peak Position eV		Area Ratio/%				
		PDA	PDA-50PY	PDA-100PY	PDA-150PY	PDA-180PY
**C1s**	C=C (248.2 ± 0.1)	26.05	27.35	27.94	23.90	18.55
	C–H_x_, C–C (285)	30.17	36.36	32.46	36.67	37.42
	C–N (286)	15.2	13.70	19.77	16.59	16.59
	C–O (286.7 ± 0.1)	11.93	8.69	6.55	9.84	10.62
	C=O (288)	14.96	13.46	12.58	12.72	14.24
	π-π* (291)	1.69	0.44	0.70	0.28	2.58
**N1s**	=N–R (398.6 ± 0.1)	16.06	14.91	19.34	18.26	7.82
	NH–R_2_ (399.9 ± 0.1)	79.79	80.04	72.10	72.57	84.75
	NH_2_–R (401.5 ± 0.1)	4.15	5.05	8.56	9.17	7.43
**O1s**	O=C (531.1 ± 0.1)	31.71	23.99	23.03	22.53	25.75
	O–C (532.8 ± 0.2)	68.29	76.01	76.97	77.47	74.25

## Data Availability

Data are contained within the article.
